# Perspective: Presuming Autistic Communication Competence and Reframing Facilitated Communication

**DOI:** 10.3389/fpsyg.2022.864991

**Published:** 2022-03-10

**Authors:** Melanie Heyworth, Timothy Chan, Wenn Lawson

**Affiliations:** ^1^Reframing Autism, Sydney, NSW, Australia; ^2^Macquarie School of Education, Macquarie University, Sydney, NSW, Australia; ^3^School of Arts and Education, Australian Catholic University, Melbourne, VIC, Australia; ^4^Department of Education, University of Birmingham, Birmingham, United Kingdom

**Keywords:** facilitated communication, rapid prompting method, complex communication needs, epistemological violence, communication competence

## Abstract

Debate surrounding the validity of the method of supported typing known as facilitated communication (FC) has been continuous since its inception in the 1990s. Views are polarized on whether FC can be considered an authenticated method for use by people with complex communication needs (CCN) or significant challenges in speech, language, and communication. This perspective article presents an analysis of the research arguing for—and against—the use of FC, combined with the lived experience knowledge of autistic adults who utilize FC, to rehabilitate its current standing as discredited and unevidenced. By considering extant qualitative and quantitative studies, as well as personal accounts of the use of this particular Augmentative and Alternative Communication (AAC) method, the authors argue that the current dismissal of FC is rooted in ableist and outdated approaches. FC research should be reconsidered and reconducted using current best practice autism research approaches, including coproduction and a presumption of autistic communication competence, to assess its validity as a potential AAC method for autistic individuals.

## Introduction

Autistic individuals with complex communication needs (CCN) are often required to rely upon Augmentative and Alternative Communication (AAC) methods for functional communication. Within the available range of AAC methods, support can be necessary to enable typing on electronic and non-electronic devices for those autistic individuals presenting with co-occurring motoric and planning/coordination challenges. Two such supported communication methods, Facilitated Communication (FC) and Rapid Prompting Method (RPM), were developed to provide greater scope and flexibility for self-expression by end-users (Crossley, 1994).

Despite an initial proliferation in the use of FC after its popularization in the 1990s, such mediated communication methods soon attracted significant debate (Green, 1994; Stock, 2011). In the decades following its inception, FC has repeatedly been decried as controversial, and discredited as unevidenced. This reaction has led to a ban on its use, echoed across peak bodies, academia, and medical and allied health professionals (e.g., American Psychological Association, 2003; International Society for Alternative and Augmentative Communication, 2014; American Speech-Language-Hearing Association, 2018).

The uncompromising dismissal of FC largely rests on the quantitative research conducted predominantly in the 1990s, when FC was in its infancy. Following the evolutionary trajectory of autism research (O’Reilly et al., 2019), the 1990s FC research was undoubtedly colored by the prevailing deficits-based ideological framework surrounding autism at the time. Three decades later, with co-production and neurodiversity beginning to shape autism research questions, design and research priorities, it is time to reassess. It is time to challenge the potential impact of research bias underpinned by ableist assumptions toward nonspeaking autistic people on our current perceptions of FC and supported communication methods.

Indeed, alongside lived experience testimonies, robust and peer-reviewed research exists to challenge a categorical anti-FC position, although, strikingly, the presence of such research has rarely been acknowledged by those who adhere to an anti-FC stance. In this Perspective piece, we argue for a re-assessment of FC. Given advances in autism research and understood within a human rights frame, all individuals have an inalienable and basic human right to self-expression through all forms of communication. That individuals are entitled to utilize their preferred means of communication is set out in the Universal Declaration of Human Rights (Article 19, UDHR; United Nations General Assembly, 1948) and the Convention of the Rights of Persons with Disability (Article 21; CRPD, United Nations General Assembly, 2008). Hence, we argue that accessing preferred means of communication for individuals with CCN should be upheld as an important avenue to self-expression and empowerment. Without this avenue, such individuals might be denied their voice and a means to achieving communicative competence for participation and autonomy.

## A Background to FC Research

Facilitated communication is a process of communication utilized by the nonspeaking disabled population, in which the communicator is supported or assisted physically by a facilitator, or communication partner (Biklen, 1990, 2000). FC was introduced initially by Crossley (1994) who described a communication partner using physical support to assist the communicator to point to pictures, words, letters, and/or numbers on a range of communication aids. Such physical assistance varies considerably depending on the FC-user’s needs, but might involve stabilizing support at the hand, wrist, forearm, elbow, or underarm, or touch or pressure on the shoulder or back, or even simply physical co-presence with no actual touching occurring. Crossley (1997) argued that with familiarization with and practice of FC, physical support could be withdrawn as appropriate to match increasing communicator proficiency. The original aim of FC, then, was to encourage individuals toward independent communication, fading physical support as soon as appropriate, not unlike the process of prompt fading that is used in other autism interventions (Cengher et al., 2018).

In model of Crossley (1994), the communication partner would ensure the necessary physical support to stabilize the user’s movement, inhibit impulsive typing, and/or to encourage the initiation of typing or pointing. Importantly, the communication partner would also give emotional, attentional, and regulatory support to encourage communication and assist the user in focusing on the keyboard, pictures, letters, or words during the communicative process. The quality of relationship between the communicator and their communication partner was paramount to the success of the communication.

Many disabled and autistic individuals with CCN received FC positively, as a new AAC method with the potential to increase their communicative competence *via* an effective and flexible communication method. Yet FC was not received so optimistically by all. Early in its development, vocal detractors raised concerns about the method’s validity. Specifically, critics questioned whether it was the user/typist or the facilitator/communication partner who authored the typed output (Schlosser et al., 2014). In the decades since, these arguments have remained ongoing, with increasing polarization between pro- and anti-FC positions (Cardinal and Falvey, 2014; Hemsley et al., 2018).

Throughout the 1990s, several published studies emerged to challenge the authenticity and authorship of communication produced through FC (e.g., Eberlin et al., 1993; Green, 1994; Bebko et al., 1996). These studies followed strict experimental procedures of message passing, a process in a controlled environment to ascertain agency under different exposure conditions, in which researchers used an object, instruction, or question prompt to solicit a response from the FC-user alone, and, separately, from the FC-user and their communication partner or facilitator. In the absence of positive results validating FC with message passing protocols, findings were interpreted as indicating the undue influence of the communication partner on the typed output, and the authorship of the output and the communication competence of the FC user were questioned. Based on the conclusion that FC messages are authored by the communication partner rather than the autistic or disabled individual themselves, FC was—and continues to be—deemed invalid and the call to prohibit its use loudly voiced (Schlosser et al., 2014; Hemsley et al., 2018).

However, naturalistic peer-reviewed journal articles, which supported FC and argued the validity of its authorship, also proliferated in the 1990s. In contrast to the comparatively few quantitative studies published in peer-reviewed journals after 1996 (Cardinal and Robledo, 2012), these have continued to be published over time. Indeed, peer-reviewed studies confirming autistic or disabled authorship of FC messages number over a hundred from the 1990s to the present (Cardinal and Falvey, 2014), and use varied methodologies including text analysis (Bernardi and Tuzzi, 2011), naturally occurring message passing (Biklen et al., 1995; Weiss et al., 1996), intensive video analysis (Emerson et al., 2001), inductive analysis (Broderick and Kasa-Hendrickson, 2001), and linguistic structural analysis (Niemi and Karna-Lin, 2002). This body of evidence speaks to the need to reassess FC, given that its validity and efficacy are not so unproblematically dismissed (Williams, 2020).

Autistic adults with CCN who utilize FC are increasingly attesting to the FC as their chosen AAC method for developing communicative competence in people with CCN (e.g., Chan and Chan, 2019; Peña, 2019). The methodology of FC is evolving to address the controversy, with a focus on the systemic development of best practice in effective use and improved techniques (Broderick and Kasa-Hendrickson, 2001; Rubin et al., 2001). Such qualitative evidence is underpinned by more quantitative approaches, including video eye-tracking. Studying an FC user’s eye gaze to verify that the autistic or disabled individual has targeted letters or letter series prior to making a hand movement toward that target, has been instrumental in providing additional support for the validity of FC-authorship (Grayson et al., 2012).

A recent naturalistic investigation utilizing sophisticated video-based eye-tracking technology, considered the real-world communication experiences of nine nonspeaking autistics who communicated by letterboard in the presence of a trained communication partner (Jaswal et al., 2020). This study authenticated the authorship of the autistic user/typist. Measuring the speed, accuracy, timing, and pattern of eye gaze fixation, the autistic participants (the letterboard users) were found to be actively typing their own thoughts, and the results negated cueing from their communication partner.

Another recent quantitative study using accelerometry, or measures of finger movement, has also provided additional evidence to confirm the authorship of autistic or disabled user/typist, signifying FC as a valid potential method of communication for non-speakers (Faure et al., 2021). In this study, the index finger of both communicator and communication partner were measured for fine motor events in typing speed, time, and acceleration produced during keystrokes. Results indicated that with a variety of physical supports, the autistic typist was found to be contributing actively to the typed output in motion acceleration toward the letters which preceded the facilitator.

Similar support has come from linguistic analysis of typed messages that reveal the communicators’ unique use of language (Zanobini and Scopesi, 2001; Niemi and Karna-Lin, 2002; Tuzzi, 2009). The body of evidence in favor of FC is thus substantive enough to warrant a reassessment of FC, with new research designs like that of Jaswal et al. (2020) and Faure et al. (2021) to utilize not only significant technological advancements to evaluate FC authorship, but also which are framed by contemporary research approaches to autism as a neurodevelopmental condition (Pellicano and den Houting, 2021) and to inclusive autism research (Fletcher-Watson et al., 2021).

## Study Design in FC Research

In FC research, as elsewhere, the perspective exists that quantitative research designs are superior (both more reliable and valid) to qualitative, interpretive, or other methodological approaches (Schlosser et al., 2014; Travers et al., 2014; Hemsley et al., 2018; Williams, 2020), resulting in a skewed picture of FC research. For example, Schlosser et al. (2014) set strict hierarchical criteria, which effectively excluded all quantitative descriptive and qualitative data from their review, resulting in “overwhelming … evidence for facilitator control” (p. 363) from the peer-reviewed, experimental, conditional design studies that the authors deemed appropriate for consideration. A 2018 review by Hemsley et al. (2018) similarly dismissed (or missed) studies that validated FC, by concluding that sufficient prior scientific evidence exists from studies prior to 2014 to prove FC is ineffectual and unauthenticated, and to support a position banning its use.

While peer-reviewed, experimental, conditional design studies have undeniable scientific validity, the authors of this Perspective argue that dismissing descriptive quantitative, qualitative and, indeed, testimonial first-person evidence ignores the imperative to understand communication holistically, within an interpersonal, social pragmatic viewpoint (Kecskes, 2010). Reducing communication to clinically measurable message passing is reductive and discounts the very real benefits that embracing a more socio-cognitive approach to communication might offer Autistics with CCN.

Importantly, when research designs have taken into consideration autistic participants’ anxiety, hypersensitivities, and related issues, and have proactively worked to alleviate those compounding factors, FC authorship has been more likely to be authenticated (Cardinal et al., 1996; Sheehan and Matuozzi, 1996; Weiss et al., 1996). The experimental designs and methodology used for investigating FC make an immense difference to the results obtained. Quantitative methods which employ strict experimental conditions in message passing, by and large, have not supported validity. Conversely, qualitative studies, and those quantitative studies, the design of which allows for the effects of environmental and participant factors, or which utilize progressive digital technology, have indicated support for authenticity and authorship of typists/users. This is unsurprising given that empirical positivist approaches, which privilege experimental, quantitative, and statistical analysis studies, in general have less explanatory power than more qualitative, interpretive approaches, which recognize the role and impact of subjectivity and intersubjectivity on what is known or assumed (Torbert, 2021).

Perhaps most disturbingly, many critiques of FC employ an antagonistic tone, which undermines “healthy scholarly engagement and … other ways of knowing” (Connor, 2019) and makes it difficult for researchers and Autistic individuals alike to engage in the kinds of robust dialog that might examine, for example, researcher bias, implicit bias, ableism, and unwillingness to embrace a presumption of communication competence (Lester, 2015). For instance, FC has been termed “pseudoscience” (Jacobson et al., 1995; Travers et al., 2014) and deemed totally without merit as a communication tool for those with CCN (Simpson and Myles, 1995; Myles et al., 1996; Travers et al., 2014). Such antipathy makes respectful debate framed by contemporary best practice approaches to autism research (Fletcher-Watson et al., 2019, 2021; den Houting et al., 2021; Keating, 2021) unlikely.

## The Issue of Research Bias

As noted, quantitative methodologies are often privileged as more scientifically valid (Mahoney and Goertz, 2006) in reviews of FC evidence, since they tend toward objective, neutral, and generalizable findings. However, we—as researchers—are (or should be) becoming more aware of the deep impact of implicit and unspoken biases (the “isms,” like racism, sexism, and ableism) on all findings and interpretations of findings. Any claims of “scientific objectivity” and “neutrality” should be thoroughly interrogated and challenged (Evans, 2002; Stanovich and West, 2008; Teo, 2010; Williams, 2020).

In FC research, we argue, it should at least be entertained that strict experimental quantitative research reflects *myside bias* (Evans, 2002; Stanovich and West, 2008). *Myside bias* refers to evidence generated from experimental designs, with data evaluated, tested, and analyzed in ways that reflect preconceptions derived from researchers’ own beliefs, opinions, and attitudes (Stanovich et al., 2013).

Facilitated communication research that suffers from *myside bias* is designed—consciously or not—to invalidate FC by adopting purely deterministic paradigms and experimental designs with a singular focus on the ability of participants to pass messages (Eberlin et al., 1993; Wheeler et al., 1993; Green, 1994; Simpson and Myles, 1995; Bebko et al., 1996; Saloviita, 2018; Vyse et al., 2019). Many FC researchers have proposed that the experimental tasks of message-passing should be the only proof required for the validity/invalidity of FC (Saloviita, 2018). This position ignores or dismisses the interpersonal pragmatic dimension of communication, and the humanity of those communicating. In so doing, many FC researchers project *myside bias* into their research design and review analysis by consistently disregarding contrary research authenticating authorship of FC users which adopts more comprehensive and naturalistic data collection methods.

Such *myside bias* is evident in the dismissal by some researchers of autistic communicators using FC who can also independently write or type (e.g., Higashida, 2013; Mukhopadhyay, 2021) or who have in time become independent of physical support (e.g., Kedar, 2012; Rubin, 2021; Sequenzia, 2021). It strikes the authors of this Perspective, that such summary rejection of the lived experience knowledge and testimonies of independent FC users is indicative not only of research bias but also perpetuates the routine silencing of the autistic “voice” in autism research (Milton and Moon, 2012).

Perhaps most importantly, many FC studies have ignored the unique developmental trajectory of nonspeaking autistics, as well as the potentially significant challenges autistic individuals with CCN might experience in clinical, experimental environments. Such challenges include high anxiety levels, hypersensitivities and sensory overload, and performance stress under unfamiliar testing conditions and in unfamiliar environments (see, Jaswal et al., 2020). Rigorous empirical evidence emerging from the 1990s onward, incorporating findings from the fields of psychoneuroimmunology (PNI), epigenetics, and bioenergetics (Segerstrom, 2012), has indicated that environmental factors, stress, and other mental states have significant negative effects on the communicative performances of nonspeaking autistics (Cardinal and Falvey, 2014). Unsurprisingly, then, studies that take such unique challenges into account and are designed to recognize communication as complex systems incorporating many elements of social interaction, are more likely to validate FC communication (Emerson et al., 2001).

## Discussion: Presumption of Communication Incompetence

Epistemology is the study of the nature and production of knowledge. Knowledge produced through empirical research comprises both research data and their interpretations according to theoretical frameworks. Various interpretations are possible in examination of data according to the types of theories used, and “knowledge” is invariably socially constructed within associated normative and cultural contexts (Teo, 2010).

Take, for example, a presumption of incompetence associated with cognitive impairment in nonspeaking autistic children, which is commonplace (e.g., Simmons et al., 2021). The communicative (in)competence and/or cognitive impairment of research participants are not inherent in data but are socially constructed in the interpretations of data according to frames of references. We would argue that these data are too often skewed by biased frames (of presumed incompetence) that distort and misrepresent individuals with CCN and exclude their perspectives. This argument is sustained by research that shows conventional measures for intelligence (e.g., WISC test batteries) generally underestimate the ability of nonspeaking autistics (Courchesne et al., 2015; Nadar et al., 2016; Akhtar and Jaswal, 2019). When individuals with CCN, who were identified as having Intellectual Disability using language-based measures, were reassessed using more appropriate non-language-based instruments that employ visual spatial tasks, a significant proportion were found to be within or above the expected IQ range (Dawson et al., 2007; Barbeau et al., 2013; Courchesne et al., 2015; Crossley and Zimmerman, 2019). The conflation of not speaking, and not having anything to say, then, is a product of neuronormative and ableist perspectives that privilege verbal communication and construct hierarchies of communication competence.

Such issues pertain to epistemological violence which emerges when interpretive knowledge is accepted as “truth,” despite the interpretative process being grounded in assumptions of inferiority and “othering” (Teo, 2010). Epistemological violence is experienced disproportionately by nonspeaking autistics because researchers and professionals claim the authority and prestige of expertise (Willis, 2020), and nonspeaking autistics are very often multiply marginalized, not least because of their CCN.

It is an unfortunate reality that there exists a prevalent presumption of incompetence affecting so called “low functioning” or “severe” autistic individuals with CCN, that is accompanied by a presumed lack of ability, desire, and capacity to communicate. Thus, some researchers and professionals accept as self-evident that, given the low level of language development of a nonspeaking autistic, any typed communication *via* FC/RPM demonstrating typical or above normal linguistic competence, cannot possibly be that individual’s output (Jacobson et al., 1995; Konstantareas and Gravelle, 1998; Simmons et al., 2021). Framed by such ableist assumptions, the voices of individuals with CCN, some of whom are independent typists, have consistently been ignored or dismissed.

Epistemological violence is evident when researchers insist that facilitator influence is the only way to account for FC/RPM users who failed message passing tasks under strict experimental conditions. This interpretation of data, we would argue, presupposes that autistic individuals with CCN lack the ability to communicate (rather than the ability to speak), although as we have noted, communicative performance is inevitably impacted by autistic challenges like anxiety, hypersensitivities, motoric differences, and being confronted with an unfamiliar environment and novel task requirements (e.g., Shoener et al., 2008).

Epistemological violence is inherent in denying the autistic “voice” authenticity of *all* output from FC users, even those who have become independent typists/writers (Vyse et al., 2019; Simmons et al., 2021). Such sweeping assertions “others” not only the FC/RPM users themselves, but also their social network, including parents and facilitators with no desire to gain from manipulating those they support (e.g., Kedar, 2012, 2018; Mukhopadhyay, 2021; Rubin, 2021).

Fundamentally, it is epistemological violence to deny nonspeaking autistics the right to self-expression and to silence their voices by denying their right to explore supported AAC (Woodfield and Ashby, 2016). It is usually claimed that the use of FC/RPM should be banned because individuals with CCN have the basic right not to be manipulated by facilitators (e.g., Simmons et al., 2021). But given that a reductive dismissal of FC has been contested by both research and nonspeaking autistics themselves, it seems prudent to re-evaluate the received position on FC so that we do not—albeit inadvertently—commit an even greater rights violation. At the very least, as researchers, we have a duty of care to acknowledge and listen to the voices of FC/RPM users who have become independent of physical support and who have irrefutably demonstrated cognitive and communicative competence (e.g., Kedar, 2012, 2018; Higashida, 2013; Mukhopadhyay, 2021; Rubin, 2021; Sequenzia, 2021).

## Conclusion

The debate surrounding the validity of FC has continued since its inception with polarized positions on FC as a valid method for communication by individuals with CCN. The existing quantitative studies (approximately 40) unsupportive of FC, identify facilitator influence within typed output, and were mostly undertaken in the 1990s. Conversely, over 100 peer-reviewed articles validate FC, and other evidence authenticating authorship exists to support the validity and efficacy of FC for autistic individuals with CCN. Perhaps most importantly, in personal narrative information using autoethnographic approaches (Ellis et al., 2011), communicative competence, agency and autonomy has been established by many nonspeaking autistic individuals, including those who once used FC.

The authors of this Perspective argue that given this evidence, our developing understanding of communication as complex processes of interpersonal, socio-cognitive, and pragmatic bidirectional exchange, moves within autism research to embrace coproduced, participatory research. Our evolving understanding of autism within a biopsychosocial model of disability and a neurodiversity framework behoves us to reconsider the standard, accepted position which dismisses and invalidates FC communications. We must consider the possibility that assisted typing is valid and offers a flexible communication tool for self-expression for certain individuals. This is not to say, of course, that all autistic individuals with CCN will benefit from FC/RPM, but that it is an inalienable human right to have the choice to access supported AAC if it is indicated for any individual.

## Author Contributions

TC, an FC-user, conceived the Perspective and researched with the support and guidance of WL and MH. TC wrote a broad argument. MH revised and prepared this Perspective, based on TC’s arguments, with iterative input from TC and WL. All authors contributed to the article and approved the submitted version.

## Conflict of Interest

The authors declare that the research was conducted in the absence of any commercial or financial relationships that could be construed as a potential conflict of interest.

## Publisher’s Note

All claims expressed in this article are solely those of the authors and do not necessarily represent those of their affiliated organizations, or those of the publisher, the editors and the reviewers. Any product that may be evaluated in this article, or claim that may be made by its manufacturer, is not guaranteed or endorsed by the publisher.
